# Intra-arterial peptide-receptor radionuclide therapy for neuro-endocrine tumour liver metastases: an in-patient randomised controlled trial (LUTIA)

**DOI:** 10.1007/s00259-023-06467-y

**Published:** 2023-10-28

**Authors:** S. C. Ebbers, M. W. Barentsz, D. M. V. de Vries-Huizing, M. W. J. Versleijen, E. G. Klompenhouwer, M. E. T. Tesselaar, M. P. M. Stokkel, T. Brabander, J. Hofland, A. Moelker, R. S. van Leeuwaarde, M. L. J. Smits, A. J. A. T. Braat, M. G. E. H. Lam

**Affiliations:** 1 Department of Radiology and Nuclear Medicine, University Medical Centre Utrecht, Heidelberglaan 100, 3584 CX Utrecht, The Netherlands; 2 Department of Radiology and Nuclear Medicine, Maxima Medical Center, Veldhoven, The Netherlands; 3 Department of Nuclear Medicine, Netherlands Cancer Institute, Amsterdam, The Netherlands; 4 Department of Radiology, Netherlands Cancer Institute, Amsterdam, The Netherlands; 5 Department of Gastrointestinal Oncology, the Netherlands Cancer Institute, Amsterdam, The Netherlands; 6 Department of Radiology & Nuclear Medicine, ENETS Centre of Excellence, Erasmus MC Cancer Institute, Rotterdam, The Netherlands; 7 Department of Internal Medicine, Section of Endocrinology, ENETS Center of Excellence, Erasmus MC Cancer Institute, Rotterdam, The Netherlands; 8 Department of Endocrine Oncology, University Medical Centre Utrecht, Utrecht, The Netherlands

**Keywords:** Neuroendocrine tumour, PRRT, Intra-arterial, Safety, Efficacy

## Abstract

**Purpose:**

Peptide receptor radionuclide therapy (PRRT) using [^177^Lu]Lu-DOTATATE has been shown to effectively prolong progression free survival in grade 1–2 gastroenteropancreatic neuroendocrine tumours (GEP-NET), but is less efficacious in patients with extensive liver metastases. The aim was to investigate whether tumour uptake in liver metastases can be enhanced by intra-arterial administration of [^177^Lu]Lu-DOTATATE into the hepatic artery, in order to improve tumour response without increasing toxicity.

**Methods:**

Twenty-seven patients with grade 1–2 GEP-NET, and bi-lobar liver metastases were randomized to receive intra-arterial PRRT in the left or right liver lobe for four consecutive cycles. The contralateral liver lobe and extrahepatic disease were treated via a “second-pass” effect and the contralateral lobe was used as the control lobe. Up to three metastases (> 3 cm) per liver lobe were identified as target lesions at baseline on contrast-enhanced CT. The primary endpoint was the tumour-to-non-tumour (T/N) uptake ratio on the 24 h post-treatment [^177^Lu]Lu-SPECT/CT after the first cycle. This was calculated for each target lesion in both lobes using the mean uptake. T/N ratios in both lobes were compared using paired-samples *t*-test.

**Findings:**

After the first cycle, a non-significant difference in T/N uptake ratio was observed: T/N_IA_ = 17·4 vs. T/N_control_ = 16·2 (*p* = 0·299). The mean increase in T/N was 17% (1·17; 95% CI [1·00; 1·37]). Of all patients, 67% (18/27) showed any increase in T/N ratio after the first cycle.

**Conclusion:**

Intra-arterial [^177^Lu]Lu-DOTATATE is safe, but does not lead to a clinically significant increase in tumour uptake.

**Supplementary Information:**

The online version contains supplementary material available at 10.1007/s00259-023-06467-y.

## Research in context

Peptide receptor radionuclide therapy (PRRT) using [^177^Lu]Lu-DOTATATE prolongs progression-free survival in patients with grade 1 and 2 midgut neuro-endocrine tumours. However, both the NETTER-1 trial and several cohort studies showed lower progression free survival in patients with bulky or extensive neuroendocrine tumour liver metastases (NELM). A few small retrospective and non-randomized studies investigated the effect of intra-arterial PRRT which is deemed to enhance tumour uptake in hepatic metastases, showing promising results. However, the true added effect of intra-arterial PRRT on efficacy and toxicity remains unclear to date.

### Added value of this study

The heterogeneity of neuroendocrine neoplasms required a randomized controlled trial to assess the true effects of intra-arterial [^177^Lu]Lu-DOTATATE. As inter-patient comparison would be compromised by tumour heterogeneity, thus an in-patient randomized controlled trial design was chosen to reduce this important bias. A significant increase in tumour uptake after intra-arterial administration is thought to result in improved tumour response and ultimately survival, thereby outweighing the increased patient burden and risks of multiple hepatic artery catheterizations.

### Implications of all the available evidence

Intra-arterial [^177^Lu]Lu-DOTATATE administration was safe, but did not result in a clinically significant increase in tumour uptake. Besides, no differences in response after three and six months were observed. Therefore, the results do not justify the intra-arterial administration of PRRT in patients with NELM.

## Background

Neuroendocrine tumours (NET) constitute a heterogeneous group of tumours. They range from well-differentiated slowly proliferative tumours to moderately differentiated rapidly growing tumours. Some tumours secrete active substances (i.e. functioning tumours), while others do not (i.e. non-functioning). Their specific characteristics together with their wide variety in localizations complicate treatment. Moreover, in 32–63% of all patients with NET, metastases are already present at the time of diagnosis [1, 2]. Irrespective of the stage of the disease, the reported overall five-year survival rates are over 60%. However, when neuroendocrine liver metastases (NELM) are present, the median overall survival decreases to 2–4 years for GEP-NET [3, 4]. Unfortunately, the vast majority of metastases are located in the liver and besides the impaired survival, patients with NELM also have a lower quality of life [5].

Intravenous administered somatostatin receptor-targeting radiopharmaceuticals, i.e. peptide-receptor radionuclide therapy (PRRT), improves progression free survival (PFS) in patients with advanced midgut NET, progressive on somatostatin analogues [6]. The radiopharmaceutical binds to the somatostatin receptor, overexpressed on the cell surface of the tumour cells, followed by internalization of the radionuclide-peptide-receptor complex. The emitted radiation damages the DNA, which subsequently leads to the induction of cell death [7]. The NETTER-1 trial randomly assigned 229 patients to receive intravenous [^177^Lu]Lu-DOTATATE plus octreotide LAR (long acting release, 30 mg every four weeks) versus octreotide LAR alone (60 mg every four weeks) [6]. PFS rate at 20 months was 65·2% versus 10·8% in favour of [^177^Lu]Lu-DOTATATE. As much as 84% of patients in NETTER-1 had NELM. While the extent of NELM did not relate to PFS, more bulky disease (i.e. target lesion > 3 cm) however did relate to a significantly decreased PFS [8]. In general, patients with (bulky) NELM have significantly shorter time to progression and suffer from increased morbidity and mortality. In the largest published cohort, including over 500 patients, it was shown that patients with extensive liver metastases have a worse outcome in terms of overall survival after treatment with [^177^Lu]Lu-DOTATATE [9].

To improve treatment of liver metastases multiple studies indicated that intra-arterial administration of radiolabelled somatostatin receptor analogues into the hepatic artery may increase the uptake in NELM [10]. Repeated [^68^Ga]Ga-DOTATOC PET/CT (interval 4 weeks) in 15 NELM patients showed a standardized uptake value (SUV) increase in 117/122 (96%) liver metastases, which was 1·44–7·eightfold higher after intra-arterial administration compared to intravenous administration [11]. This finding was confirmed in a preclinical animal study and a subsequent pilot study in three patients with NELM using ^111^In-DTPA-octreotide with an increased tumour uptake up to 2·ninefold after intra-arterial administration [12, 13].

The aim of our study was to treat patients with bulky NELM, with an indication for [^177^Lu]Lu-DOTATATE PRRT, by an intra-arterial approach via a microcatheter in the hepatic artery, instead of intravenously. Using an innovative prospective study design with in-patient randomization to guarantee effective comparison with minimal risk of bias due to heterogeneity, the additional benefit of intra-arterial over intravenous administration was investigated.

## Methods

### Study design and participants

**Fig. 1:**
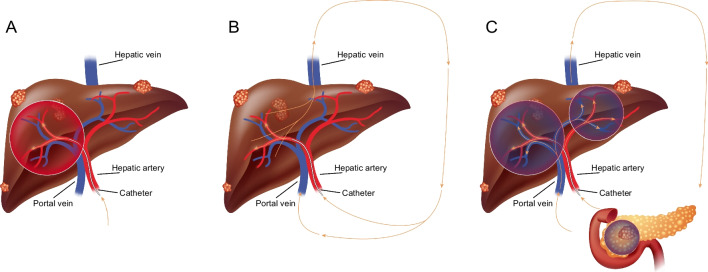
**A** Intra-arterial administration was performed in either the left or the right hepatic artery, thereby exposing half of the liver to high concentration [^177^Lu]Lu-DOTATATE. **B** [^177^Lu]Lu-DOTATATE enters systemic circulation via the hepatic vein. **C** tumours in the contra-lateral lobe and extrahepatic disease are treated via systemic circulation, both via portal vein and hepatic artery (second-pass effect)

The Lutetium Intra-Arterial (LUTIA) study is a multi-centre, open-label, phase II, within-patient randomized controlled trial (RCT). The study protocol was previously published and was approved by the medical ethics committees (ClinicalTrials.gov Identifier: NCT03590119) [14]. The study sought to investigate whether uptake of [^177^Lu]Lu-DOTATATE in liver metastases could be enhanced by intra-arterial administration as opposed to conventional intravenous administration. The study used a within-patient comparison design, in order to increase statistical power and reduce the influence of inter-patient differences on the primary outcome of the study, especially to avoid bias by tumour heterogeneity. Patients were treated intra-arterially, receiving [^177^Lu]Lu-DOTATATE (Lutathera®, Novartis) in either the left or the right hepatic artery for all four treatment cycles using 7·4 GBq per cycle. The control lobe and extrahepatic lesions received [^177^Lu]Lu-DOTATATE via a second-pass effect (Fig. 1). As primary endpoint, ^177^Lu uptake in the intra-arterially treated liver metastases was compared to control metastases in the contralateral liver lobe after the first cycle. As secondary endpoints, tumour uptake of ^177^Lu after all cycles, difference in tumour response, and toxicity were assessed. The study was performed at the University Medical Centre Utrecht (Utrecht, The Netherlands), the Erasmus Medical Centre (Rotterdam, The Netherlands) and the Netherlands Cancer Institute (Amsterdam, The Netherlands).

Patients with NELM were included according to the European Neuroendocrine Tumour Society (ENETS) guidelines: patients had an indication for [^177^Lu]Lu-DOTATATE treatment as determined by the multidisciplinary tumour board, somatostatin receptor (SSTR)-positive tumours on [^68^Ga]Ga-SSTR PET imaging, and sufficient organ function (bone marrow, liver and kidney) [15]. In addition, included patients needed to have at least one metastasis of at least 3 cm in each liver lobe. Only patients with grade 1 or grade 2 gastroenteropancreatic (GEP-)NET were included. The extent of extrahepatic metastatic disease was not an exclusion criterion. Patients with previous liver targeted therapies within one year prior to screening and patients who had undergone previous cycles with radionuclide therapy were excluded from participation.

At baseline, patient characteristics, complaints, disease characteristics, and current and previous treatment schedules were recorded. Laboratory blood tests were taken for determination of baseline hematologic, renal, and hepatic function. Finally, multiphase contrast-enhanced CT (CECT) of the abdomen and [^68^Ga]Ga-SSTR PET/CT were performed. PET/CT imaging was performed according to local acquisition protocols.

### Procedures

Prior to the first treatment, patients were randomized to receive treatment in the left or right hepatic artery. On the day of treatment, patients were admitted and had laboratory testing prior to their transfer to the angiography suite. Patients received an amino acids solution consisting of 2.5% Lysine/2.5% Arginine in 1L saline infused over 4 h, starting 30 min before start of [^177^Lu]Lu-DOTATATE infusion. Via transradial or transfemoral access, a microcatheter was placed in the designated hepatic artery. Subsequently, a cone beam CT was performed to accurately define the perfusion volume of the intra-arterially treated artery. Finally, [^177^Lu]Lu-DOTATATE was administered by flushing the [^177^Lu]Lu-DOTATATE vial with 200 mL saline during 30 min (Utrecht and Amsterdam sites) or by syringe pump injection during 30 min, followed by flushing during 15 min (Rotterdam site), after which the access site was closed by either an air-filled wrist band or femoral closure device. Patients remained admitted for one night, according to Dutch radiation safety guidelines.

**Fig. 2:**
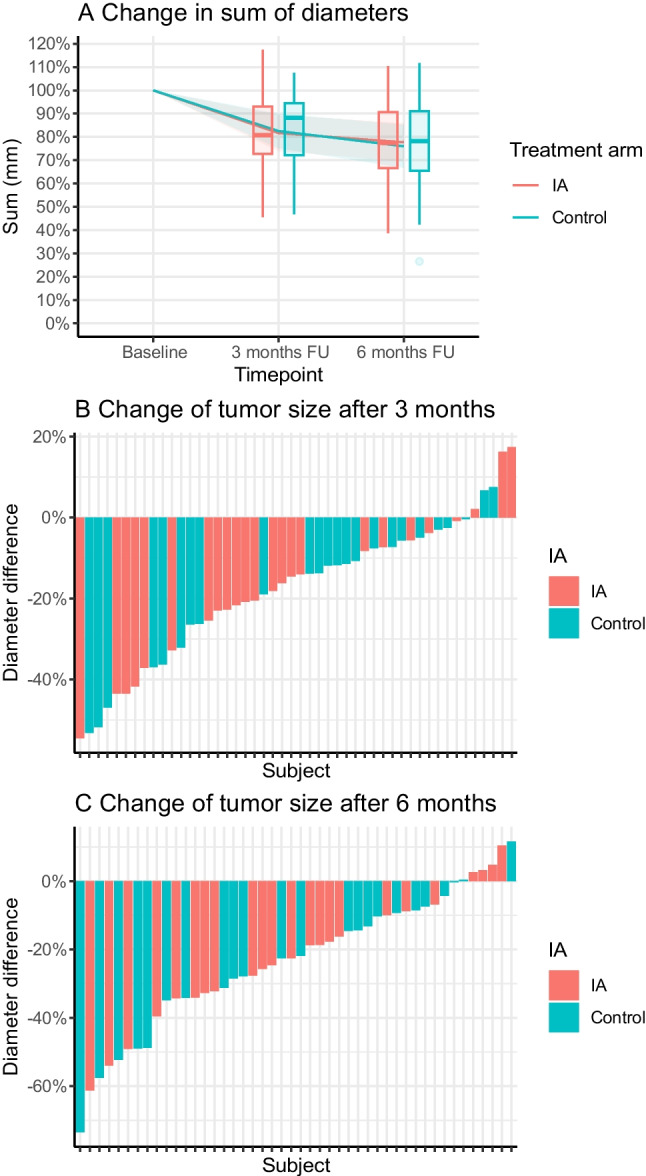
Example procedure in a 64 years old male patient with grade I small-intestinal NET, with lymphatic, hepatic, pulmonary, orbital and skeletal metastases. The patient was randomized to intra-arterial PRRT infused from the left hepatic artery. **A**: baseline CECT depicting extensive bi-lobar disease; **B**: baseline [^68^Ga]Ga-DOTATOC PET/CT maximum intensity projection depicting extensive metastatic disease; **C**: cone-beam CT showing the microcatheter placed in the left hepatic artery, and hypervascular tumours in the left liver lobe; **D**: post-treatment ^177^Lu SPECT/CT, showing similar activity distribution compared to the baseline [^68^Ga]Ga-DOTATOC PET/CT. The resulting T/N_IA_ ratio was 9.4, and the T/N_control_ ratio was 6.9. Both liver lobes showed stable disease at 3 and 6 months post-treatment follow-up imaging

Twenty-one to 27 h post-injection, ^177^Lu total body planar scintigraphy and single photon emission computed tomography/computed tomography (SPECT/CT) imaging of the liver were acquired for primary endpoint assessment (Fig. 2). Images were acquired with a Medium Energy Low Penetration (MELP) collimator. Acquisition settings were harmonized across all systems: body contour trajectory, a photon energy window of 208 keV (± 10%) and 113 keV (± 10%), adjacent 20% lower scatter windows, 2 × 64 projections, a 128 × 128 matrix size, and a reference projection time of 20 s. Four weeks after each [^177^Lu]Lu-DOTATATE cycle, adverse events, laboratory testing and eligibility for the next treatment cycle was checked at the out-patient clinic. Laboratory testing, CECT of the abdomen, and [^68^Ga]Ga-DOTATOC PET/CT were repeated at 3 and 6 months after the fourth [^177^Lu]Lu-DOTATATE cycle.

### Image analysis

Both tumour uptake and response were analysed using predefined target lesions. On baseline CECT, up to three target lesions were selected within each liver lobe, measuring at least 3 cm in diameter on axial plane CECT. A Volume of Interest (VOI) accounting for at least 15 mL was drawn in the healthy liver tissue in the control lobe (without metastasis on CECT and without pathological uptake on [^68^Ga]Ga-SSTR PET) to determine the normal uptake. VOI’s of pre-defined target lesions were segmented semi-automatically on baseline [^68^Ga]Ga-SSTR PET/CT images, by drawing a rough VOI around the target lesions, and shrinking the VOI using a threshold of 42% of the maximum voxel SUV in the initial VOI [16]. Baseline [^68^Ga]Ga-SSTR PET/CT images were co-registered to all ^177^Lu SPECT/CT images (rigid registration), and VOIs were transferred to extract the mean and peak uptake values in target lesion VOIs and in the normal-tissue VOI. Uptake was calculated as counts per voxel on SPECT/CT. Peak uptake was defined as the mean counts per voxel in a sphere-shaped VOI with a diameter of 1 cm around the voxel with the highest voxel value [17].

### Endpoints

The primary endpoint was the tumour-to-non-tumour (T/N) ratio of uptake values in target lesions between the intra-arterial (T/N_IA_) and control (T/N_control_) liver lobe on [^177^Lu]Lu-DOTATATE SPECT/CT after the first cycle. T/N was defined as the weighted average of activity uptake in the predefined VOIs, divided by the uptake in the normal tissue VOI in the control lobe.

As a secondary endpoint, T/N ratios were calculated using the peak uptake in the predefined VOIs (supplemental material). Additionally, T/N ratios of all cycles were evaluated (i.e. 18–24 h post-treatment ^177^Lu-SPECT/CT after every cycle). Objective response of pre-defined target lesions was determined on CECT at baseline, and at 3- and 6-months post-treatment, according to Response Evaluation Criteria In Solid Tumours (RECIST) version 1.1 [18]. Target lesions could be a composite of multiple confluent tumours.

Safety analysis consisted of laboratory testing (hepatic, renal and haematological parameters) and recording of clinical adverse events (AEs) during all visits. Laboratory values were categorized using Common Terminology Criteria for Adverse Events (CTCAE) version 5.0, and baseline laboratory tests were recorded. Toxicity due to PRRT was only considered if the CTCAE grade was higher than the baseline toxicity grade. Technical success rate was defined as the fraction of treatments allowing intra-arterial administration of [^177^Lu]Lu-DOTATATE.

As a post hoc analysis, factors influencing tumour uptake were tested against T/N results.

### Randomization and blinding

Patients were randomized 1:1 between intra-arterial [^177^Lu]Lu-DOTATATE via the left or the right hepatic artery using a computer-generated permuted block sequence with block sizes of *n* = 1 and *n* = 2. No stratification was used due to the small study sample. Patients and medical personnel were not blinded for the randomization result. Blinded image analysis for primary and secondary endpoints was performed (i.e. without knowledge of intra-arterial treatment lobe).

### Statistical analysis

The minimum number of patients needed to test for a moderate to large effect (i.e. a Cohen’s *d*_*z*_ of 0·65) was calculated. A power of 0·9 was achieved in case of a minimum sample size of 26 patients. All patients who received at least one cycle intra-arterially were included in statistical analysis. For the primary outcome a paired samples *t*-test was used, comparing the T/N_IA_ against T/N_control_, which is equivalent to a one-sample *t*-test on the within-patient differences in T/N. As secondary outcome, the proportional difference in T/N was tested by performing a paired samples *t*-test on the log-transformed data. Estimates (mean and 95% confidence interval (CI)) were then transformed back by taking the exponent of the log-transformed estimates. Differences in T/N, when considering all cycles, were tested using linear mixed-effects models by incorporating a random intercept on two levels, i.e. per patient (to adjust for the correlation of data within a patient) and per cycle (to incorporate the paired-samples design of the study). Factors influencing T/N were tested by adding them to a linear-effects model as co-variates. Differences in response after treatment were tested using Fisher’s exact test.

## Results

**Fig. 3:**
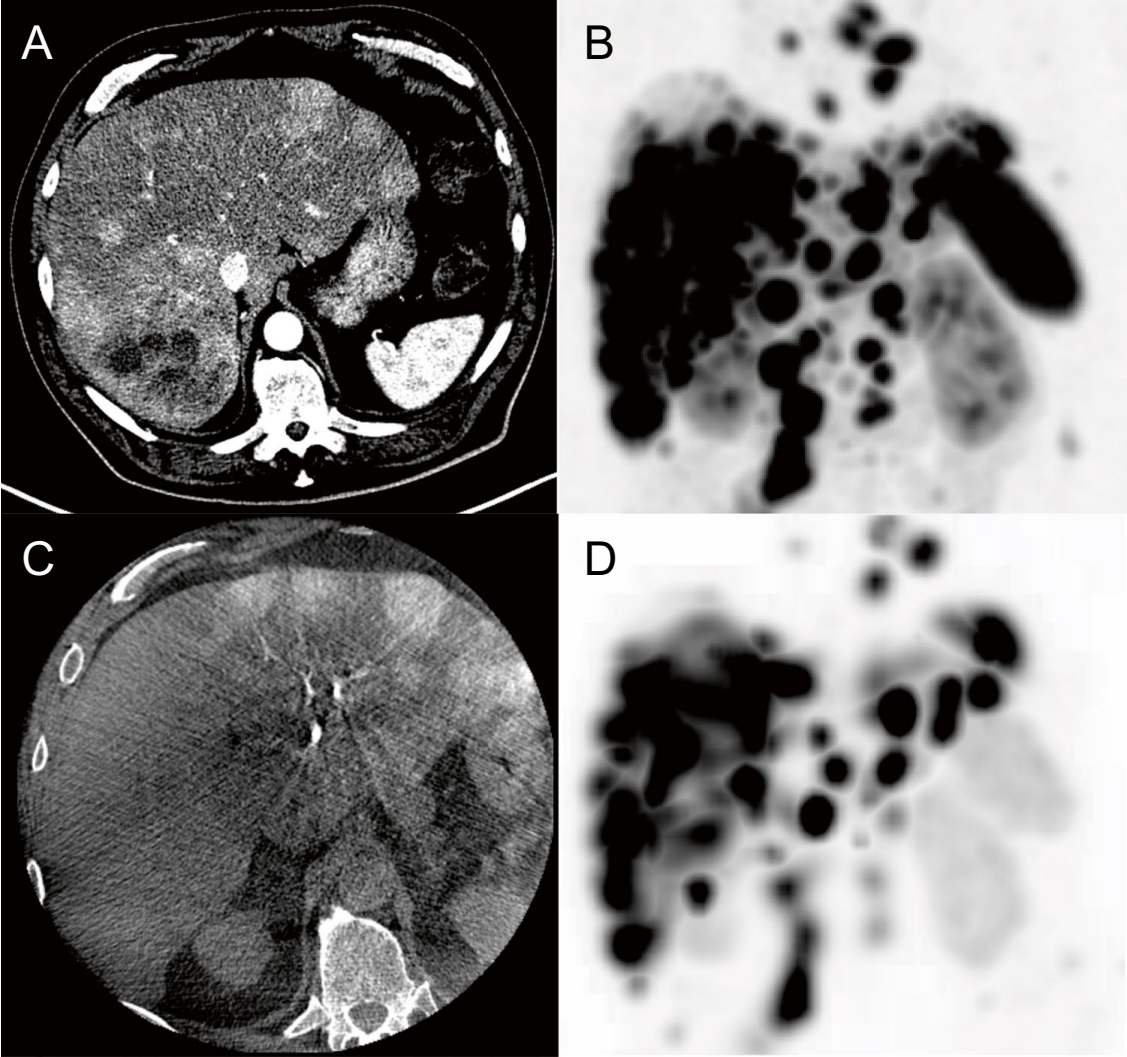
Flowchart of patient inclusion, treatment and analysis. Due to the in-patient randomization, no dedicated flowchart per treatment arm can be shown. One patient died due to disease progression after receiving the first cycle. One patient was referred for additional treatment before finishing four cycles of intra-arterial PRRT, and another patient was referred after the 3-month follow-up imaging. One patient voluntarily withdrew from the study after the first cycle. Twenty-four patients finished all four treatment cycles

In total, 30 patients were included between August 2018 and January 2022, of whom 27 patients were treated according to the study protocol. All 27 patients were included in the primary endpoint analysis. One patient was excluded and switched over to intravenous PRRT due to delivery issues of [^177^Lu]Lu-DOTATATE on the day of the scheduled intra-arterial therapy, and two patients received intravenous PRRT due to the COVID-19 outbreak. Fourteen patients (52%) received intra-arterial [^177^Lu]Lu-DOTATATE in the right hepatic artery and 13 patients (48%) in the left hepatic artery (Fig. 3). There were no crossover events. Technical success rate was 100%. Two patients only completed one cycle, of which one voluntarily withdrew from the study, and one patient died due to disease progression. One patient was referred for additional treatment after receiving two cycles due to disease progression. Baseline characteristics are reported in Table 1.

**Table 1:** Baseline characteristics

	Median (range) or Count (%)
Total number of patients	*27*
Male gender	20 (74%)
Age	63 (42–76)
ECOG-PS	
0	19 (70%)
1	8 (30%)
Months since diagnosis	11·4 (0·7–109)
Primary tumour origin	
Pancreas	11 (41%)
Small bowel	10 (37%)
Colon	1 (4%)
Rectum	1 (4%)
Unknown	4 (15%)
Tumour grade	
1	6 (22%)
2	21 (78%)
Ki67 index	7 (1–18)
Previous treatments	
Somatostatin analogues	20
Everolimus	1
Months of follow-up	13 (1·3–15)
Completed cycles of PRRT	
1	2 (7%)
2	1 (4%)
4	24 (89%)

Baseline characteristics of all patients included in the primary endpoint analysis

*ECOG-PS* Eastern Cooperative Oncology Group Performance Score

### Primary endpoint

**Fig. 4:**
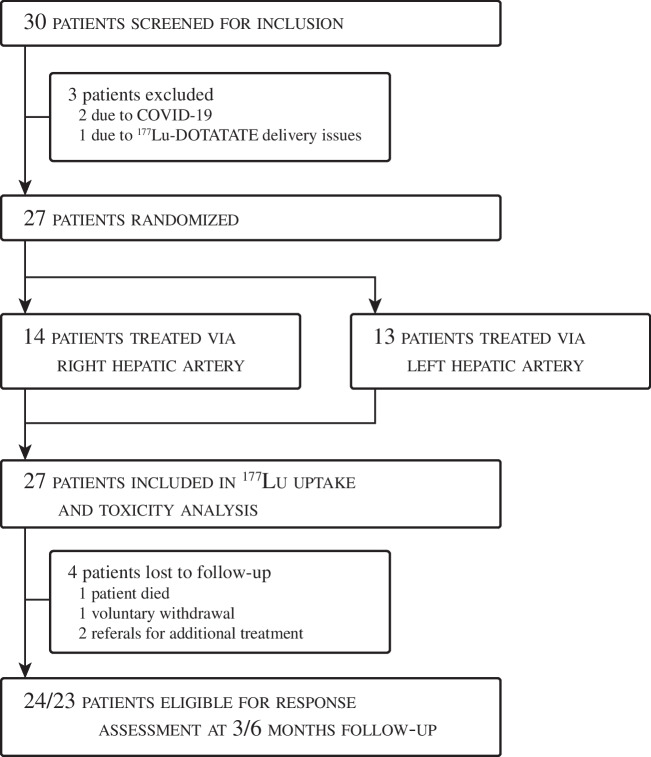
**A** Boxplot of the T/N uptake ratios observed in control and intra-arterially treated liver metastases, calculated on the 24 h post-treatment SPECT/CT after the first cycle (primary endpoint). **B** Uptake in intra-arterially treated liver metastases relative to control tumours. Mean relative change = 17% (*p* = 0·045, 95% CI [0·00, 0·31]). T/N = Tumour-to-non-tumour; IA = Intra-arterial

There was no significant difference between the mean uptake-based T/N_IA_ and T/N_control_ ratios on SPECT/CT after the first cycle, with a mean T/N_IA_ of 17·9 (95% CI [10·6, 25·1]) and a mean T/N_control_ of 16·2 (95% CI [8·2, 24·2], *p* = 0·299). The mean difference between T/N_IA_ and T/N_control_ was 1·65 (95% CI [-1·55, 4·85]; Fig. 4). A total of 18/27 patients (67%) had a higher T/N_IA_, than T/N_control_. Five patients (19%) had an increase in T/N_IA_ of more than 50%, and two patients (7%) had a T/N_IA_ increase of more than 100%.

### Secondary endpoints

After the first cycle, the relative difference between T/N_IA_ and T/N_control_ was 0·17, i.e. the T/N_IA_ was 17% higher on average than the T/N_control_, (*p* = 0·045, 95% CI [0·00, 0·31]). One-hundred post-treatment ^177^Lu SPECT/CT scans acquired in 27 patients were available for analysis of secondary endpoints. There was no significant difference between the T/N_IA_ and T/N_control_ when all cycles are combined, with a mean T/N_IA_ of 13·5 (95% CI [11·0, 16·0]) and a mean T/N_control_ of 12·4 (95% CI [9·7, 15·2], *p* = 0·216). The absolute mean difference was 1·03 (95% CI [-0·61, 2·68]). The T/N_IA_ was 12·7% higher compared to T/N_control_, when considering the multiplicative difference (*p* = 0·031, 95% CI [0·01, 0·26]). In addition to the mean uptake in each VOI, the peak uptake was used in secondary analysis. After the first cycle, and after all cycles, the absolute increase in peak uptake was not statistically significant (*p* = 0·091 and *p* = 0·637, respectively). However, a small significant relative increase was found both after the first cycle and after all cycles (*p* = 0·010 and *p* = 0·045, respectively; Supplemental 1). The following possible factors of influence tested insignificant: whether tumours were visually hypervascular (*p* = 0·217); the liver lobe that was selected for intra-arterial administration (*p* = 0·713); the hepatic tumour burden (*p* = 0·053).

**Fig. 5:**
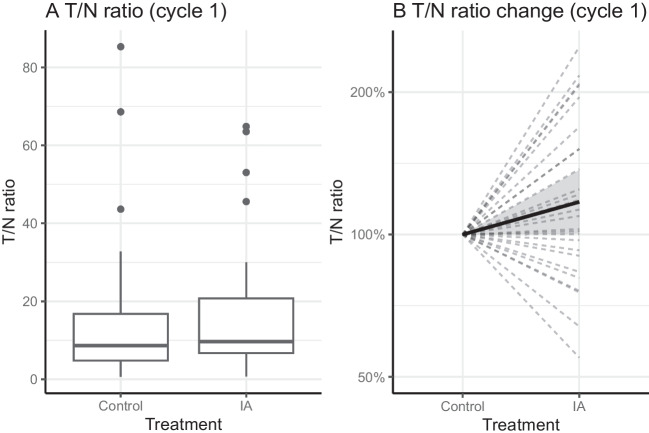
Response of hepatic metastases after intra-arterial treatment. **A**: trend of mean diameter of liver metastases with confidence interval from baseline to 6 months post-treatment. **B** and **C**: Waterfall plots showing the relative change in diameter of all target lesions in each liver lobe compared to baseline. Intra-arterially treated lobes and control lobes are distributed rather uniformly across the plot, indicating no significant difference in response. Note: each subject is present in each waterfall plot twice, due to intra-patient comparison. IA = Intra-arterial

Response was assessed in 24 patients 3 months after treatment and in 23 patients 6 months after treatment in whom post-treatment CECT was available. One patient was referred for additional treatment before imaging was performed after 6 months follow-up. At 3 months after the final cycle, partial tumour response was seen in 6/24 (25%), while stable disease was observed in the remaining 18/24 (75%) cases in both intra-arterially treated liver lobes and control lobes (*p* = 1·0) (Fig. 5). At 6 months post-treatment, there were 8/23 (35%) cases with partial response, 14/23 (61%) with stable disease, and 1/23 (4%) with progressive disease. Again, no difference in tumour reduction was found (*p* = 1·0) (Table 2).

**Table 2:** Response at 3 and 6 months follow-up

	Control	Intra-arterial	*p*-value
3 months			1·0
PR	6 (25%)	6 (25%)	
SD	18 (75%)	18 (75%)	
6 months			1·0
PR	8 (35%)	8 (35%)	
SD	14 (61%)	14 (61%)	
PD	1 (4%)	1 (4%)	

Response categories of liver lobes (i.e. control and intra-arterially treated) on CECT at 3 and 6 months post treatment according to RECIST 1.1. Differences tested with Fisher’s exact test

*PR* partial response; *SD* stable disease; *PD* progressive disease

**Fig. 6:**
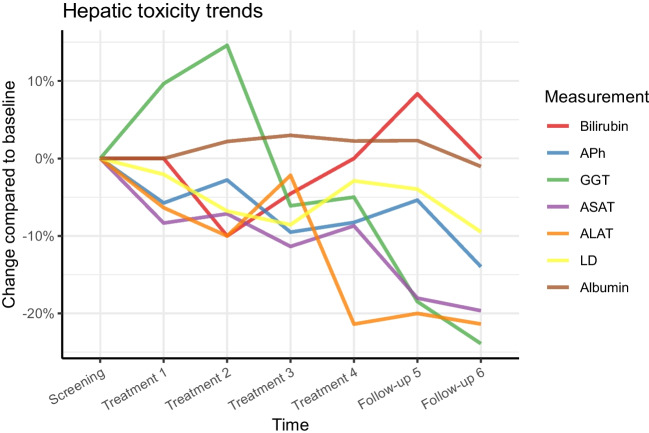
Mean hepatic parameters during follow-up after intra-arterial administration of [^177^Lu]Lu-DOTATATE show a slight decrease of pre-existent elevated liver enzymes and no indication of additional hepatic toxicity

Toxicity was acceptable and as expected (Table 3). In the 26·4 patient-years of follow-up, two patients experienced CTCAE grade 3 or 4 hepatic toxicities: one patient with baseline gamma-glutamyl transferase (γ-GT) elevation grade 3 and baseline alkaline phosphatase (ALP) elevation grade 2, temporarily developed grade 4 γ-GT elevation and grade 3 ALP elevation, followed by an almost complete normalization of γ-GT and ALP after observing partial tumour response. Another patient with baseline γ-GT elevation grade 2 developed grade 3 toxicity due to intrahepatic disease progression. Overall, average liver enzyme trend lines showed no change greater than 15%, with a slight decrease in liver enzyme levels during follow-up (Fig. 6). Concerning haematological toxicity, grade 3 lymphocytopenia was observed in 7 (26%) of patients, and grade 4 lymphocytopenia was observed in 1 (4%) patient. No other grade 3–4 hematologic toxicity was observed and no renal toxicity was encountered.

**Table 3:** Biochemical toxicity during follow-up

CTCAE grade	1	2	3	4
Hepatic				
Albumin	2			
ALAT	5			
ASAT	6	1		
γ-GT	3	5	1	1
ALP	4		1	
Bilirubin	2			
Hematologic				
Hemoglobin	11	3		
MCV	10	4		
Thrombocytes	13	2		
Leukocytes	11	4		
Neutrophiles	4	2		
Lymphocytes		10	7	1
Renal				
eGFR	8	2		
Creatinin	2			
Urea	11			

Number of patients with experienced biochemical toxicity, in which the highest observed toxicity during follow-up was recorded. Total number of patients is 27

*ALAT* alanine aminotransferase; *ASAT* aspartate aminotransferase; *γ-GT* gamma-glutamyl transferase; *ALP* alkaline phosphatase; *MCV* mean corpuscular volume; *eGFR* estimated glomerular filtration rate

During follow-up, multiple clinical AEs were encountered (Table 4). The most common AEs were grade 1 and grade 2 fatigue (37% and 33%) and grade 1 and grade 2 nausea (37% and 11%). In most patients, fatigue occurred in the weeks following PRRT, while nausea occurred shortly after administration despite pre-treatment with anti-emetics. Nine patients (33%) complained of pain, swelling or hematoma at the femoral or radial puncture site. However, no complications requiring intervention were observed. Two patients had light back pain following the angiographic procedure, while one patient complained of severe back pain. One patient suffered a moderate allergic reaction to iodine contrast agent at the third angiography procedure, and one patient suffered a severe allergic reaction to iodine contrast agent during the acquisition of a follow-up CECT. Unrelated to the angiography, one patient was hospitalized for a carcinoid crisis twice, 11 days and 10 days after the first and the fourth treatment cycle, respectively.

**Table 4:** Clinical toxicity during follow-up

Adverse Event	Grade 1	Grade 2	Grade 3	Grade 4	Grade 5
Fatigue	10	9			
Nausea	10	3			
Puncture site complaints	9				
Abdominal pain	5	3	1		
Diarrhoea	5	1			
Vomiting	5				
Decreased appetite	4				
Flushing	3	2			
Back pain	2		1		
Hair loss	2				
Headache	2				
Sweating	2				
Xerostomia	2	1			
Allergic reaction		1	1		
Cholangitis			1		
Fracture			1		
GI bleeding			1		
Gastritis			1		
Infection			1		
Urinary tract infection			1		
Carcinoid crisis				1*	
Disease progression					1
Other	11	5	1		

Clinical adverse events graded according to CTCAE 5.0 occurring during follow-up after intra-arterial [^177^Lu]Lu-DOTATATE. *Occurred twice in the same patient

## Discussion

A non-significant increase in tumour uptake of [^177^Lu]Lu-DOTATATE of 17% was found after intra-arterial administration compared to systemic administration. Furthermore, no difference was found in objective response rates between intra-arterial and intravenous treated liver metastases during the first 6 months after treatment. Besides angiographic procedure related side effects, no additional toxicity was found, but the lack of clinical benefit does not seem to justify an intra-arterial treatment approach in this setting.

Using a within-patient randomization design, many in-between patient biases were avoided. A moderately-large effect size was deliberately chosen, because sufficient patient benefit should be gained to compensate for the additional risks (e.g. bleeding, artery dissection) and burden of multiple angiography procedures. Due to the high statistical power, it is unlikely that a clinically significant increase in tumour uptake will be found by increasing the study sample. This is supported by the secondary endpoint analysis, in which a small but statistically significant increase in T/N_IA_ of only 12.7% was found. This minor non-significant increase in T/N_IA_ did not result in an increased tumour response within 6 months after the fourth treatment.

Other groups have previously studied the effects of intra-arterial PRRT, using different radiopharmaceuticals, all based on the same hypothesis: an increase in tumour uptake in NELM might be achieved by intra-arterial administration, as exposure to high concentration of a radiopharmaceutical during a short period of time may induce complete saturation of SST_2_ receptors. In a previously published review, multiple retrospective studies showed predominantly favourable outcomes after intra-arterial administration. Three studies by Kratochwil et al. are the most noteworthy [10]. In one study, 15 patients were injected with [^68^Ga]Ga-DOTATOC intravenously and intra-arterially (i.e. into the main hepatic artery), with 4 weeks in-between, reporting a 3.75-fold increase in SUV-measurements after intra-arterial administration [11]. However, injection-to-imaging delay, affinity, dosing, and specific activity differ significantly between [^68^Ga]Ga-DOTATOC and [^177^Lu]Lu-DOTATATE, so that an increase in uptake using other radiopharmaceuticals cannot be directly inferred. This was already described in a letter to the editor by Brogsitter et al. [19]. In a follow-up study, 15 patients were treated intra-arterially with a combination of [^177^Lu]Lu-DOTATOC and [^90^Y]Y-DOTATOC, in which a response rate of 60% was reported [12]. In a substudy in the same study population, four patients underwent dynamic scintigraphy while receiving intra-arterial and intravenous administration of ^111^In-DOTATOC, which showed significant wash-out after intra-arterial administration. An increased retention of activity in tumours was found at 1, 4, 24, 48 and 72 h after injection, but with diminishing differences over time. The same washout was shown by Pool et al., in three patients after intra-arterial administration of ^111^In-DTPA-octreotide [13]. More recently, Lawhn-Heath et al. treated patients with G1-3 NET intra-arterially with a single cycle of [^90^Y]Y-DOTATOC [20]. The study was halted after treating 10 patients, due to the minimal treatment efficacy as determined by the comparison of response rate of treated liver lesions with data from the NETTER-1 study. In five patients, co-injection of [^68^Ga]Ga-DOTATOC in the hepatic artery was performed, followed by PET/CT imaging and quantitative analysis and comparison with intravenous [^68^Ga]Ga-DOTATOC administration, which showed no significant difference in uptake in hepatic metastases either. However, competition of both used compounds for the SSTR may have resulted in differences in uptake between [^68^Ga]Ga-DOTATOC and [^90^Y]Y-DOTATOC, complicating the interpretation of their results. In another study, Thakral et al. treated 15 patients with intra-arterial PRRT using [^177^Lu]Lu-DOTATATE and compared the uptake in liver metastases with 14 patients who were treated intravenously. A threefold increase in T/N ratio from 25.6 to 78.5 between the two groups was reported [21]. Patient selection and treatment arm allocation however were not reported. Furthermore, differences between the two groups in terms of disease status, histopathology, previous treatments, and extrahepatic disease were unclear, while these factors may have significant impact on tumour uptake. These studies suggested, to a greater or to a lesser extent, that intra-arterial PRRT may increase tumour dose. The currently presented study shows no difference in tumour uptake after intra-arterial injection of [^177^Lu]Lu-DOTATATE.

In the LUTIA study, four aspects may have contributed to the limited increase in T/N_IA_. First, the affinity for the SST_2_ receptor of [^177^Lu]Lu-DOTATATE is by itself extremely high. Therefore, no increase in SSTR binding proportion can really be expected by increasing the concentration [22]. Second, subsequent internalization of the SST_2_-[^177^Lu]Lu-DOTATATE complex is fast and efficient (within minutes), and intracellular trapping of [^177^Lu]Lu-DOTATATE lasts for several hours, significantly reducing binding sites on the outside of NELM tumour cells [23]. Therefore, [^177^Lu]Lu-DOTATATE is possibly mostly extracted from systemic circulation, instead of during first pass. Third, due to the highly vascularized nature of both the liver and metastases, circulatory exposure to [^177^Lu]Lu-DOTATATE is already high. Finally, the peptide mass in the [^177^Lu]Lu-DOTATATE solution administered differs depending on manufacturing procedures and may result in differences in the bound proportion of receptors, as part of the solution will consist of peptides not bound by radioactive ^177^Lu. These “cold” peptides still populate some of the SST_2_ receptors, preventing “hot” peptides (i.e. bound by ^177^Lu) to be bound and internalized by the neuroendocrine tumour cell, resulting in a lower tumour uptake [24]. When a solution with a low specific activity is used, there are less SST_2_ receptors available for binding with “hot” peptides, limiting differences between intra-arterial or systemic exposure.

Besides these conceptual causes, the absence of a significant increase in tumour uptake, may also be partially attributable to a high variance in T/N ratios. In the current study population, T/N ratios varied between patients and within patients. Even using a within-patient study design, this high variance could not be completely removed. Some of the variance is explained by a small unavoidable mismatch between baseline CT and post-treatment SPECT/CT. No factors could be identified associated with a higher uptake ratio in our patients, as a cause for the remaining variance. The liver lobe that was treated intra-arterially, the visually hypervascular appearance, nor the hepatic tumour burden were significantly associated with a change in T/N ratio. Furthermore, within-patient tumour heterogeneity in NELM is well-known, with approximately 40% of patients having different tumour grades in-between different metastases [25, 26].

There are some shortcomings to the current study. Firstly, the study design using in-patient comparison of intra-arterial versus intravenous administration does not allow for accurate estimation of true activity uptake in the control lobe, as the pharmacokinetics may differ when compared to regular intravenous application. Secondly, the study group was rather heterogeneous in terms of primary tumour, time since diagnosis, and liver involvement. Therefore, the power of the study was insufficient to show efficacy in subgroup analyses. Thirdly, it is unknown whether the 24 h timepoint is the best timepoint for measuring the uptake. Different results could be found when choosing a different timepoint, and cumulative activity cannot be directly inferred from the reported measurements. However, a sub study with multiple-timepoint imaging was performed for dosimetric analyses, of which the analyses are still running. Lastly, one specific commercially available radiopharmaceutical compound was used (i.e. Lutathera®, Novartis). A different outcome may be possible when other radiopharmaceuticals are used, for example with different amounts of peptide.

There are still some challenges concerning intra-arterial PRRT that need to be addressed. First, as previously mentioned, different radiopharmaceuticals may be studied to show differences in tumour uptake after intra-arterial administration. This may be due to difference in peptide concentration, SST_2_ affinity, or differences in pharmacokinetics. Secondly, intra-arterial administration of [^177^Lu]Lu-DOTATATE may be feasible when considering other indications besides NET, such as meningioma [27]. Other methods are available to enhance the effect of PRRT, for example with hepatic radioembolization. This combination was already shown to be both effective and safe when using ^166^Holmium microspheres in the HEPAR PLuS study [28, 29]. Other potential combinations may include systemic treatments, as radiosensitizers or inhibiting DNA-repair mechanisms [30].

In conclusion, intra-arterial administration of [^177^Lu]Lu-DOTATATE in grade 1 to 2 NET yields a minimal non-significant increase in tumour uptake, without any effect on tumour response. This does not justify the intra-arterial administration of [^177^Lu]Lu-DOTATATE in the patients with NELM.

### Supplementary Information

Below is the link to the electronic supplementary material.

Supplementary file1 (DOCX 147 KB)

## Data Availability

The datasets generated during and/or analysed during the current study are available from the corresponding author on reasonable request.
