# Supplementary material for: Intra-arterial peptide-receptor radionuclide therapy for neuro-endocrine tumour liver metastases: an in-patient randomised controlled trial (LUTIA)
Source: Eur J Nucl Med Mol Imaging. 2023 Oct 28;51(4):1121–32. doi: 10.1007/s00259-023-06467-y (PMC10881701; doi:10.1007/s00259-023-06467-y)
Supplement: Supplementary file 1 — Supplementary file1 (DOCX 147 KB) [file 259_2023_6467_MOESM1_ESM.docx]

# Supplemental material

## S1

|  | Control lobe | IA lobe | *p for absolute increase* | Mean increase | *p for relative increase* |
| --- | --- | --- | --- | --- | --- |
| Peak uptake (1^st^ cycle) | 32·5 | 38·0 | *0·091* | 23% | *0·010* |
| Peak uptake (all cycles) | 28·3 | 29·5 | *0·637* | 13% | *0·045* |

Results of secondary analysis of uptake measurements using the peak uptake in predefined VOI’s. A sphere with a diameter of 1 centimetre around the voxel with the highest voxel value in each VOI. Both the analysis using only the data from the first treatment cycle, and the analysis using all cycles are presented.
